# Supplementing the diet of Nile tilapia (*Oreochromis niloticus*) with microalgae *Nannochloropsis oculata* and *Phaeodactylum tricornutum* enhanced immune function, lipid profile, and resistance to *Edwardsiella tarda* infection

**DOI:** 10.1007/s11259-026-11218-z

**Published:** 2026-04-18

**Authors:** Domickson Silva Costa, Jucimauro de Araújo Pereira Júnior, Paola Capistrano dos Santos, Maria Clara Miguel Libanori, Gracienhe Gomes dos Santos, Ana Paula de Souza, Alexandre Vaz da Silva, Cláudia Andrea Lima Cardoso, Arlene Sobrinho Ventura, Aline Brum Figueredo Ruschel, Rafael Sales, Fábio de Farias Neves, Marco Shizuo Owatari, Maurício Laterça Martins, Scheila Anelise Pereira Dutra, Cláudio Manoel Rodrigues de Melo

**Affiliations:** 1 Marine Molluscs Laboratory (LMM), Aquaculture Department, Federal University of Santa Catarina (UFSC), Florianópolis, SC Brazil; 2 Marine Shrimp Laboratory (LCM), Aquaculture Department, Federal University of Santa Catarina (UFSC), Florianópolis, SC Brazil; 3 Aquatic Organisms Health Laboratory (AQUOS), Aquaculture Department, Federal University of Santa Catarina (UFSC), Florianópolis, SC Brazil; 4 Federal Institute of Santa Catarina (IFC), Araquari, SC Brazil; 5 Center for Studies in Natural Resources, State University of Mato Grosso do Sul (UEMS), Dourados, MS Brazil; 6 Faculty of Agricultural Sciences (FCA), Federal University of Grande Dourados (UFGD), Dourados, MS Brazil; 7 Marine Fish Farming Laboratory (LAPMAR), Aquaculture Department, Federal University of Santa Catarina (UFSC), Florianópolis, SC Brazil; 8 Algabloom, Laguna, SC Brazil; 9 Algae Cultivation and Biotechnology Laboratory (LCBA), State University of Santa Catarina (UDESC), Laguna, SC Brazil

**Keywords:** Aquaculture, Feed additives, Fish immunity, Phycobiostimulants

## Abstract

The aim of the present study was to evaluate the effects of dietary supplementation with the microalgae *Nannochloropsis oculata* and *Phaeodactylum tricornutum*, either separately or in combination, on growth performance, lipid profile, haematoimmunological parameters, and resistance to *Edwardsiella tarda* infection in Nile tilapia (*Oreochromis niloticus*). A total of 960 juvenile Nile tilapia (2.87 ± 0.68 g and 5.65 ± 0.16 cm) were allocated to 24 experimental units (*n* = 40) and fed six experimental diets for 60 days: DC_0%_ (no microalgae inclusion), DN_0.5%_ (0.5% *N. oculata*), DN_1%_ (1% *N. oculata*), DP_0.5%_ (0.5% *P. tricornutum*), DP_1%_ (1% *P. tricornutum*), and DPN_1%_ (0.5% *N. oculata* + 0.5% *P. tricornutum*). After the feeding period, fish were challenged with *E. tarda* (2 × 10⁸ CFU mL⁻¹). Microalgal inclusion increased significantly (*p* < 0.05) dietary contents of phenolic compounds, flavonoids, tannins, and antioxidant potential of diets without affecting fish growth. The DP_1%_ and DPN_1%_ diets increased significantly (*p* < 0.05) eicosapentaenoic and docosahexaenoic acid levels and reduced monounsaturated fatty acids and ∑saturated fatty acids in muscle, thereby improving the lipid profile and flesh quality. Following infection, reductions in erythrocytic parameters were observed, along with increases in lymphocyte, monocyte, basophil, neutrophil, and thrombocyte counts, as well as higher (*p* < 0.05) agglutination and antimicrobial titres, total plasma protein, and immunoglobulin levels in the DP_1%_ and DPN_1%_ groups. Survival was higher in the microalgae-supplemented groups, particularly in DPN_1%_ (91.67 ± 1.8%) compared with the control (65.0 ± 2.0%). Supplementation with DP_1%_ and DPN_1%_ improved haematoimmunological responses, lipid composition, and resistance to *E. tarda* in Nile tilapia.

**Supplementary Information:**

The online version contains supplementary material available at 10.1007/s11259-026-11218-z.

## Introduction

Nile tilapia (*Oreochromis niloticus*) is a tropical freshwater species widely produced in several regions of the world, contributing significantly to the economies of many countries. In 2023, global production of this species reached 5.5 million tonnes, consolidating tilapia as the second most widely produced fish species worldwide (FAO 2025). Nevertheless, as Nile tilapia production increases, challenges to animal health also intensify. High stocking densities and the presence of pathogens, particularly bacteria, represent significant challenges to tilapia production. Disease outbreaks typically occur as a result of an imbalance among the components of the host–pathogen–environment triad (Wang et al. 2023).

Bacterial diseases are particularly significant due to their diversity, opportunistic nature, and potential lethality, depending on the host’s health status and the bacterial load in the culture environment. Bacterioses are caused by a range of aetiological agents, including *Aeromonas hydrophila*, *Streptococcus iniae*, *S. agalactiae*, *Francisella orientalis*, and *Edwardsiella tarda*, which are commonly associated with major outbreaks and economic losses in tilapia farming (Cavalcante et al. 2020; Wang et al. 2020; He et al. 2021). Among these pathogens, *E. tarda* deserves particular attention, as it is an emerging bacterium in the aquaculture sector and is also associated with zoonotic infections (Algammal et al. 2022; Okasha et al. 2025).

*E. tarda* is a Gram-negative, rod-shaped bacterium that causes edwardsiellosis, also known as septicaemic bacterial disease, a severe condition that frequently results in high mortality in farmed fish (Korni et al. 2021; Elgendy et al. 2022). This pathogen is highly virulent and affects a wide range of freshwater fish species, including Nile tilapia (Elgendy et al. 2022; Goh et al. 2023). Infection with *E. tarda* presents visible clinical signs such as exophthalmia, erratic swimming, haemorrhage, and ascites, as well as microscopic lesions including splenic and hepatic congestion and renal enlargement (Pandey et al. 2021; Guo et al. 2022; Goh et al. 2023).

To combat the spread of this disease, antibiotics are frequently used in fish farms for both prophylactic and therapeutic purposes. Nevertheless, the excessive and inappropriate use of these medications, sometimes carried out illegally, contributes to the development of antibiotic-resistant bacterial strains and so-called superbugs (Oh et al. 2020). In addition, residues of these drugs adversely affect the environment and the fish themselves, posing a threat to public health (Manzoor et al. 2023). Consequently, sustainable alternatives for disease control and management in aquaculture have been extensively investigated.

Various organic substances, including plant-derived compounds (phytobiotics) (Kari et al. 2022; Goh et al. 2023) and microorganism-based products (probiotics) (Ghosh 2025), have been recognised for their effectiveness in maintaining health in aquaculture. In recent years, biocompounds derived from macro- and microalgae (phycobiotics) (Bahi et al. 2023; Sattanathan et al. 2024; Tawfeek et al. 2024) have gained increasing attention in this context. When administered at appropriate doses, these compounds have been shown to enhance antimicrobial, antioxidant, and anti-inflammatory capacities in aquatic organisms. For instance, Sattanathan et al. (2024) reported that supplementing the diet of tilapia (*O. mossambicus*) with *Chaetomorpha aerea* at a rate of 5 g kg⁻^1^ enhanced fish immunity, thereby improving their ability to resist infections. Similarly, Charoonnart et al. (2018) highlighted the nutritional benefits of microalgae as a supplement in aquafeeds, citing their high digestibility and rich content of protein, lipids, and essential nutrients.

Indeed, microalgae are particularly beneficial for fish species due to their ability to boost immune function, improve resistance to diseases, and enhance tolerance to environmental stress (Bahi et al. 2023). Microalgae also exhibit diverse biochemical properties, including antioxidant, anticoagulant, antimicrobial, and antitumour activities (Yang et al. 2021; Mousavian et al. 2022). In this regard, microalgae like *Nannochloropsis oculata* (Zahran et al. 2021) and *Phaeodactylum tricornutum* (Reis et al. 2021a, b; Celi et al. 2022) are considered promising options for use in aquaculture.

*N. oculata* is characterised by high levels of chlorophyll, long-chain polyunsaturated fatty acids (ω-3 PUFAs), including eicosapentaenoic acid (EPA), as well as flavonoids, alkaloids, glycosides, β-carotene, and phenolic compounds (Sales and Santos 2020; Zahran et al. 2021; Salem et al. 2022). Abdelghany et al. (2020) and Salem et al. (2022) reported that the inclusion of *N. oculata* at 50–100 g kg⁻¹ and 100–150 g kg⁻¹ in the diet of *O. niloticus* increased resistance against *Aeromonas veronii* and *A. hydrophila*, modulated the immune system, and improved growth performance and antioxidant enzyme activity, respectively.

Regarding the diatoms, *P. tricornutum* contains fucoxanthin, EPA, β-glucans, and other PUFAs (Reis et al. 2021a, b), as well as docosahexaenoic acid (DHA) (Jovanovic et al. 2021; Kadalag et al. 2022). Its biochemical composition comprises approximately 36.4% protein, 26.1% carbohydrates, and 18.0% lipids (Celi et al. 2022), in addition to compounds that possess antimicrobial properties (Yang et al. 2023). Additionally, when incorporated into the culture of aquatic organisms, *P. tricornutum* can exert beneficial effects (Duarte et al. 2021) and may be used as a prophylactic measure against stress-related events, such as bacterial diseases (Reis et al. 2021a, b). It has also demonstrated antiparasitic activity against monogeneans in *Poecilia reticulata* following 24 h immersion baths (Jawaji et al. 2023), as well as anti-inflammatory effects in zebrafish (*Danio rerio*) fed *P. tricornutum* isolates (Samarakoon et al. 2023). The functional effects of microalgae on fish have been extensively investigated and are well established. However, uncertainties remain regarding the inclusion of different microalgal species, either individually or in combination, in Nile tilapia diets.

In this context, the present study aimed to evaluate the effects of incorporating the microalgae *N. oculata* and *P. tricornutum* into the diet of juvenile Nile tilapia, either separately or in combination, on growth performance, lipid profile, health, and survival following exposure to *E. tarda*.

## Materials and methods

### Biological material

The Nile tilapia GIFT strain (all-male monosex) were obtained from Acqua Sul^®^ fish farm, Ilhota, Santa Catarina, Brazil. The microalgae *N. oculata* and *P. tricornutum* were cultivated, lyophilised, and supplied by Algabloom^®^, Laguna, Santa Catarina, Brazil. The *E. tarda* strain (ATCC 15947) was isolated from infected fish, deposited at the Fisheries Institute (São Paulo, SP), and provided by the Laboratory of Aquatic Organism Health (AQUOS) at the Federal University of Santa Catarina. All animal handling procedures were approved by the Ethics Committee on Animal Use of the Federal University of Santa Catarina (CEUA/UFSC 6551240523).

### Experimental diets

Feed preparation was adapted from Heluy et al. (2023), using lyophilised biomass of the microalgae *N. oculata* and *P. tricornutum*, which were incorporated into a commercial basal diet (without additives) for omnivorous fish. The feed was ground using a Wiley-type knife mill, after which the microalgae were added at two inclusion levels (5 and 10 g kg⁻¹), with water at 55 °C added to promote agglutination. The mixture was homogenised in a horizontal mixer until a semi-solid consistency was obtained and subsequently pelletised using a die with a 2.5 mm aperture. The pellets were dried in a forced-air oven at 28 °C for 4 h, fragmented, and sieved to obtain granules ranging from 2.0 to 2.5 mm in size. The diets were then stored at − 20 °C until use.

The proximate composition of the diets following microalgal inclusion (Supplementary file 1) was determined according to the methods established by AOAC (2016). Crude protein (*N* × 6.25) was determined using the Kjeldahl method, whereas crude lipid content was assessed using a Soxhlet extractor with petroleum ether as the solvent. Crude fibre was determined by acid and alkaline digestion of the samples, following the procedures described by Van Soest (1991) and Silva and Queiroz (2002).

### Bioactive compounds of the experimental diets

Bioactive compounds were determined using 1.0 g of sample extracted with 10 mL of ethanol for 28 min under ultrasound, followed by filtration and analysis. This procedure was applied to all experimental diets and to the pure microalgal biomass. For the quantification of phenolic compounds, 0.1 mL of the extract was added to 0.5 mL of Folin–Ciocalteu reagent and 1.0 mL of distilled water, followed by one minute of incubation. Subsequently, 1.5 mL of sodium carbonate (20%) was added, and the absorbance was measured using a spectrophotometer (Global Trade Technology^®^, Brazil) at 430 nm (Djeridane et al. 2006). Quantification was performed using a gallic acid calibration curve, and the results were expressed as mg of gallic acid equivalents (GAE) per g of sample.

For flavonoid determination, 1.0 mL of aluminium chloride (2%) in methanol was added to 1.0 mL of the sample. After 15 min of reaction, the absorbance was measured using a spectrophotometer at 430 nm (Djeridane et al. 2006). Quantification was performed using an analytical calibration curve, and the results were expressed as mg of rutin equivalents (RE) per g of sample.

Tannin content was determined using the Folin–Denis spectrophotometric method (Pansera et al. 2003), with adaptations to the reagent volumes without altering their proportions. Briefly, 0.5 mL of Folin–Denis reagent was added to 0.5 mL of the sample, mixed, and allowed to stand for 3 min. Subsequently, 0.5 mL of 0.75 M sodium carbonate was added, the mixture was homogenised, and the reaction was allowed to proceed for 2 h in the dark. The absorbance was measured at 725 nm. Tannin concentration was calculated using a tannic acid standard curve, and the results were expressed as mg of tannic acid equivalents (TAE) per g of sample.

The antioxidant activity was evaluated using the DPPH (2,2-diphenyl-1-picrylhydrazyl) free radical method described by Capanoglu et al. (2008). For each 0.1 mL sample, 2 mL of a 0.004% DPPH solution was added, the reaction was allowed to proceed for 30 min in the dark, and the absorbance was measured using a spectrophotometer at 517 nm. The percentage inhibition was calculated according to equation:

1 $$\:Q=\:\frac{A0-AC}{A0}X\:100$$

where *Q* corresponds to the percentage of inhibition, *A₀* is the absorbance of the control, and *Ac* is the absorbance of the sample after the reaction.

### Experimental design

The fish were acclimatised to the experimental conditions for 10 days. During this period, they were fed a commercial diet. After acclimatisation, a total of 960 juvenile Nile tilapia, with a mean initial weight of 2.87 ± 0.68 g and a mean initial length of 5.65 ± 0.16 cm, were distributed into 24 experimental units, each with a usable volume of 80 L (*n* = 40 fish per tank), and fed six experimental diets with four replicates per treatment. For 60 days, the fish received the following diets: DC_0%_ (no microalgae inclusion), DN_0.5%_ (0.5% *N. oculata*), DN_1%_ (1% *N. oculata*), DP_0.5%_ (0.5% *P. tricornutum*), DP_1%_ (1% *P. tricornutum*), and DPN_1%_ (0.5% *N. oculata* + 0.5% *P. tricornutum*). Feeding management was conducted according to Silva and Marchiori (2018), which takes into account water temperature and fish size. Biometric measurements were performed every 15 days to monitor growth and adjust feed amounts, and excess solids were removed from the tanks twice daily by siphoning.

The experimental units were connected to a recirculating aquaculture system (RAS) with mechanical and biological filtration, ultraviolet disinfection, and a 12-hour photoperiod (Owatari et al. 2018). Water quality parameters, including pH, dissolved oxygen (DO), total ammonia (TAN = NH_3_ + NH_4_^+^), toxic ammonia (NH_3_), and nitrite (NO_2_^–^), were measured using the Labcon Test^®^ colorimetric kit (Brazil), and temperature was measured with a digital thermometer. During the experimental trial, these parameters remained within the safe ranges for Nile tilapia, with pH 6.5 ± 0.28; DO 5.88 ± 0.95 mg L⁻¹; TAN 1.6 ± 1.0 mg L⁻¹; toxic ammonia 0.011 ± 0.005 mg L⁻¹; nitrite 0.73 ± 0.50 mg L⁻¹; and temperature 26.62 ± 0.95 °C. At the end of the 60 days supplementation period, fish had a mean weight and length of 57.93 ± 2.6 g and 14.93 ± 1.74 cm, respectively. A total of 168 fish were sampled for fatty acid, haematological, and immunological analyses, corresponding to seven fish per tank, of which five were used for haematological and immunological analyses and two for fatty acid analysis.

### Growth performance

To determine the growth performance of Nile tilapia, the following indicators were used: weight gain (WG), daily weight gain (DWG), specific growth rate (SGR), feed conversion (FC), and survival (S%). These indices were calculated using the following equations:

2 $$\:WG\:\left(g\right)=\left(average\:final\:weight\:-\:average\:initial\:weight\right)$$

3 $$\:DWG\:\left(g\right)=\left[\frac{(average\:final\:weight\:-\:average\:initial\:weight)}{cultivation\:\mathrm{d}\mathrm{a}\mathrm{y}\mathrm{s}}\right]$$

4 $$SGR\: (\%\:day^{-1})=\left[\frac{(Ln(final\:weight)-Ln(initial\:weight)}{cultivation\:days}\right]\times100$$

5 $$\:FC=\left[\frac{\left(consumed\:feed\right)}{(\mathrm{f}\mathrm{i}\mathrm{n}\mathrm{a}\mathrm{l}\mathrm{w}\mathrm{e}\mathrm{i}\mathrm{g}\mathrm{h}\mathrm{t}\:-\:\mathrm{i}\mathrm{n}\mathrm{i}\mathrm{t}\mathrm{i}\mathrm{a}\mathrm{l}\:\mathrm{w}\mathrm{e}\mathrm{i}\mathrm{g}\mathrm{h}\mathrm{t})}\right]$$

6 $$\:SR\:\left(\%\right)=\left(\frac{Final\:population}{Initial\:population}\right)\times\:100$$

### Fatty acids

At the end of the 60-day experimental period, two fish per tank were euthanised using a eugenol solution (100 mg L⁻¹), followed by medullary dissection. A total of 48 muscle samples (8 per treatment) were collected and stored at − 80 °C for further analysis. Subsequently, ether extract was determined according to the method described by Bligh and Dyer (1959), using a mixture of three solvents: chloroform–methanol–water (ratio 1:2:0.8, respectively). Each sample was initially homogenised with methanol and chloroform to form a single phase. Additional volumes of chloroform and water were then added to induce separation into two distinct phases: an organic phase (chloroform) containing lipids, and an aqueous phase (methanol and water) containing non-lipid components. The organic phase was carefully isolated, and after complete evaporation of chloroform, the lipid fraction was quantified gravimetrically. The total lipids were then subjected to esterification to obtain fatty acid methyl esters (FAMEs), which were separated by gas chromatography (GC-2010 Plus, Shimadzu, Japan). Fatty acids were identified by comparing the retention times of sample peaks with those of analytical standards. A total of 37 fatty acid methyl ester standards from the FAME Mix (Sigma-Aldrich^®^) were used for identification. Quantification was performed by area normalisation, and results were expressed as the relative percentage of peak area.

### Haematoimmunological analyses

At the end of the 60-day supplementation period and following the experimental infection, five fish per tank were anaesthetised using a eugenol solution (75 mg L⁻¹), resulting in a total of 240 samples (20 per treatment), of which 120 were collected before and 120 after the bacterial challenge. Blood was collected via caudal vessel puncture using a 3.0 mL syringe containing 10% ethylenediaminetetraacetic acid (EDTA) as an anticoagulant (Ranzani-Paiva et al. 2025). Blood smears were prepared in duplicate and stained with May–Grünwald–Giemsa–Wright (Ranzani-Paiva et al. 2025). Differential leukocyte counts were performed under oil immersion (100×), with at least 200 leukocytes classified per smear, and results were expressed as relative percentages. Total leukocyte and thrombocyte counts were estimated indirectly. In each smear, 2,000 cells (including erythrocytes, leukocytes and thrombocytes) were counted, and the numbers of leukocytes and thrombocytes were recorded. Absolute values were calculated using a proportional method based on the total cell count obtained with a Neubauer chamber. The absolute number of each leukocyte type was determined by multiplying its relative percentage by the total leukocyte count, as described by Ranzani-Paiva et al. (2025). Aliquots of blood were used to determine haematocrit (Hct) (Goldenfarb et al. 1971) and total erythrocyte (RBC) counts in a Neubauer chamber after 1:200 dilution in modified Dacie solution, according to Blaxhall and Daisley (1973). Haemoglobin (Hb) concentration was determined using the cyanomethemoglobin method, adapted from Drabkin and Austin (1935). Mean corpuscular volume (MCV), mean corpuscular haemoglobin (MCH), and mean corpuscular haemoglobin concentration (MCHC) were calculated using the equations described by Ranzani-Paiva et al. (2025). Blood glucose levels were determined in situ using a portable glucometer (Accu-Chek^®^, Roche, Switzerland), according to the methods described by Bartoňková et al. (2016) and Ball and Weber (2017).

The remaining blood from the haematological analyses, collected before and after the experimental infection, was centrifuged at 1,400 × g for 15 min at 4 °C to obtain plasma. Plasma samples were pooled from five fish per tank, resulting in a total of 48 samples (4 per treatment), of which 24 were collected before and 24 after the experimental infection. Samples were stored at − 20 °C for subsequent analyses. The plasma antimicrobial titre against *E. tarda* was determined in flat-bottomed 96-well microplates according to the method described by Silva et al. (2009), using samples collected before the challenge (after 60 days of feeding) and after the experimental infection. The *E. tarda* inoculum was cultured in Brain Heart Infusion (BHI) broth for 24 h at 28 °C and diluted in poor broth (PB) to 2 × 10⁸ CFU mL⁻¹. Plasma was diluted 1:3 in PB in the first well (50 µL plasma: 150 µL PB), followed by serial 1:2 dilutions up to the 12th well. For positive and negative controls, saline solution was diluted in PB in the same manner as the plasma. Finally, 20 µL of *E. tarda* was added to wells containing diluted plasma and the positive control. Microplates were incubated for 24 h at 28 °C, and microbial growth was measured using a microplate reader at 550 nm. The antimicrobial titre was defined as the reciprocal of the last dilution showing complete inhibition of microbial growth.

The agglutination titre, as well as the antimicrobial titre, was determined before and after the experimental infection. For this purpose, the assay was performed in 96-well U-bottom microplates. Plasma was diluted 1:1 in phosphate-buffered saline (PBS) in the first well (50 µL PBS:50 µL plasma), followed by twofold serial dilutions (1:2) up to the 12th well. For the positive and negative controls, saline solution was diluted in PBS using the same procedure applied to the plasma samples. Then, 50 µL of *E. tarda* inactivated with 10% formalin was added to all wells containing diluted plasma and the positive control. Plates were incubated at 25 °C for 18 h in a humidified chamber. Agglutination was confirmed by the presence of a precipitate at the bottom of the well and was expressed as the reciprocal of the last dilution showing agglutination (Silva et al. 2009).

Total plasma protein (TPP) concentration was measured using a commercial total protein kit (Lab Test^®^). Total immunoglobulin (Ig) was determined according to the method of Amar et al. (2000). In this procedure, 100 µL of plasma was added to 100 µL of 12% polyethylene glycol (PEG) solution (Sigma-Aldrich^®^, USA) and incubated at room temperature (24 °C) for 2 h to precipitate immunoglobulin molecules. The precipitate was then sedimented by centrifugation at 5,000 × g for 10 min at 4 °C (Amar et al. 2000). The total protein content of the supernatant was measured using the commercial kit (Lab Test^®^), with bovine serum albumin used to construct the standard curve. Total immunoglobulin concentration was expressed in mg mL⁻¹ and calculated as Total immunoglobulin = Total plasma protein − Protein remaining after PEG treatment.

### *Edwardsiella tarda* challenge

The experimental infection dose was determined according to the method described by Reed and Muench (1938). For this purpose, 40 fish were distributed into four tanks (*n* = 10 per tank) equipped with heaters to maintain the water temperature at 27.0 ± 1.0 °C. Ten fish per tank were intraperitoneally (i.p.) injected with the following bacterial concentrations: 2 × 10⁸ CFU mL⁻¹, 2 × 10⁹ CFU mL⁻¹, and 2 × 10¹⁰ CFU mL⁻¹, whereas the control group received only sterile saline solution (0.65% SSS).

*E. tarda* was cultured in BHI broth at 28 °C for 24 h and then streaked onto Mueller–Hinton agar plates containing 5% defibrinated sheep blood to enhance strain pathogenicity, followed by incubation at 28 °C for 24 h. The inoculum was then cultured again in BHI under the same conditions. After growth, the inoculum was centrifuged at 4,000 × g for 30 min at 4 °C and resuspended in 10 mL of 0.65% SSE at doses of 2 × 10⁸, 2 × 10⁹, and 2 × 10¹⁰ CFU mL⁻¹, according to a prior cell growth curve. All fish received 100 µL i.p. of the respective doses. Cumulative mortality was monitored over 96 h.

At the bacterial dose of 2 × 10¹⁰ CFU mL⁻¹, all fish died within 12 h, while the 2 × 10⁹ CFU mL⁻¹ dose resulted in 90% mortality over 96 h. Both doses were therefore excluded from the experimental challenge. The dose selected for the challenge was 2 × 10⁸ CFU mL⁻¹, which caused lower mortality after 96 h according to Kaplan–Meier analysis (*p* < 0.05) (Supplementary file 2). After determining the appropriate infection dose and following the 60-day feeding period, 20 fish per tank from each experimental diet were challenged with Edwardsiella tarda at 2 × 10⁸ CFU mL⁻¹ via intraperitoneal injection (100 µL per fish). Mortality was monitored daily for 15 days, and the bacterium was re-isolated to confirm it as the causative agent.

### Statistical analyses

Data on bioactive compounds in the diets, growth performance indices, fatty acids, and mortality after the experimental challenge were subjected to one-way analysis of variance (ANOVA), while other data collected before and after the experimental infection were analysed using two-way factorial ANOVA. Data were analysed using a generalised linear model (GLM) (Nelder and Wedderburn 1972) with the glm package in R software (version 4.4.0), applying distribution functions. The variables of weight gain, daily weight gain, specific growth rate, feed conversion, survival rate, haemoglobin, haematocrit, mean corpuscular volume, glucose, total leukocytes, thrombocytes, lymphocytes, monocytes, saturated fatty acids, and antioxidant potential were modelled using a Gamma distribution. Mean corpuscular haemoglobin, mean corpuscular haemoglobin concentration, erythrocytes, polyunsaturated fatty acids, total proteins, and tannins were modelled using an inverse Gaussian distribution, while neutrophils, basophils, monounsaturated fatty acids, antimicrobial and binding titre, phenolic compounds, and flavonoids were modelled using a Gaussian distribution. Distribution functions were selected based on the Akaike Information Criterion (AIC) and residual analysis. When necessary, means were compared using Tukey’s post hoc test. The significance level was set at 5%.

## Results

### Phenolic compounds, flavonoids, tannins, and antioxidant potential

A consistent pattern was observed, in which the control diet (DC_0%_) exhibited the lowest concentrations (*p* < 0.05) of all compounds evaluated, regardless of diet composition. In contrast, the DP1% diet was distinguished by significantly higher concentrations (*p* < 0.05) of phenolic compounds, flavonoids, and antioxidant potential, with values of 34.93 ± 0.06 mg g⁻¹, 35.97 ± 0.15 mg g⁻¹, and 14.30 ± 0.10%, respectively. Tannin content was significantly higher (*p* < 0.05) in the DPN_1%_ and DP_1%_ diets, with values of 1.57 ± 0.06 mg g⁻¹ and 1.53 ± 0.06 mg g⁻¹, respectively (Table 1).

**Table 1:** Analysis of bioactive compounds and antioxidant potential (mean ± standard deviation) in the experimental diets and microalgae, including a control diet without microalgae inclusion (DC_0%_); a diet containing 10 g kg⁻¹ of *Nannochloropsis oculata* (DN_1%_); a diet containing 5 g kg⁻¹ of *N. oculata* (DN_0.5%_); a diet containing 10 g kg⁻¹ of *Phaeodactylum tricornutum* (DP_1%_); a diet containing 5 g kg⁻¹ of *P. tricornutum* (DP_0.5%_); and a diet containing 5 g kg⁻¹ of *P. tricornutum* + 5 g kg⁻¹ of *N. oculata* (DPN_1%_)

Treatments	Parameters (mg g^− 1^)			
	Phenolic compounds	Flavonoids	Tannins	Antioxidant potential (%)
DC_0%_	20.37 ± 0.06^c^	11.23 ± 0.12^c^	1.03 ± 0.06^c^	1.03 ± 0.05^c^
DN_1%_	29.97 ± 0.12^ab^	29.13 ± 0.12^b^	1.47 ± 0.06^ab^	9.53 ± 0.06^b^
DN_0.5%_	25.20 ± 0.10^b^	24.27 ± 0.06^b^	1.27 ± 0.06^b^	6.37 ± 0.04^b^
DP_1%_	34.93 ± 0.06^a^	35.97 ± 0.15^a^	1.53 ± 0.06^a^	14.30 ± 0.10^a^
DP_0.5%_	30.03 ± 0.06^ab^	30.03 ± 0.12^ab^	1.23 ± 0.06^b^	10.77 ± 0.06^b^
DPN_1%_	27.57 ± 0.06^b^	33.47 ± 0.07^ab^	1.57 ± 0.06^a^	11.60 ± 0.10^ab^
*p-*value	< 0.001	< 0.001	< 0.001	< 0.001
Antioxidant potential (mg g^–1^)				
*N. oculata*	156. 23 ± 0.12	99.63 ± 0.21	10.23 ± 0.06	63.23 ± 0.15
*P. tricornutum*	198.17 ± 0.25	112.12 ± 0.15	11.37 ± 0.06	83.43 ± 0.06

Note: Different letters ^(a, b, c)^ within the same column indicate significant differences according to Tukey’s post hoc test

### Growth performance

After 60 days of dietary supplementation, no statistically significant differences (*p* > 0.05) were observed among the treatments in tilapia growth performance (Table 2).

### Fatty acids

It was observed that the saturated fatty acid (SFA) profile in tilapia muscle was influenced by the diets. Arachidic acid (C20:0), heneicosanoic acid (C21:0), and behenic acid (C22:0) were present at significantly higher concentrations (*p* < 0.05) in the DP_0.5%_ group, whereas stearic acid (C18:0) was significantly higher (*p* < 0.05) in the DC_0%_ group. Furthermore, total saturated fatty acids (∑SFA) were significantly higher (*p* < 0.05) in the muscle of fish fed the DC_0%_ diet (44.89 ± 0.17%) compared with the other groups.

Regarding monounsaturated fatty acids (MUFAs), only palmitoleic acid (C16:1) exhibited significantly higher levels (*p* < 0.05) in the DN*0.5%* and DP_0.5%_ groups, diverging from the pattern observed for the other MUFAs. Myristoleic acid (C14:1), heptadecenoic acid (C17:1), and oleic acid (C18:1) were significantly higher (*p* < 0.05) in the DC*0%* group. Total MUFA content (∑MUFAs) was significantly lower (*p* < 0.05) in the DPN_1%_ group, whereas the DC_0%_ group showed the highest levels (31.95 ± 0.04% and 32.56 ± 0.10%, respectively).

For polyunsaturated fatty acids (PUFAs), α-linolenic acid showed a significantly higher concentration (*p* < 0.05) in the DPN_1%_ and DN_1%_ diets (1.61 ± 0.05% and 1.59 ± 0.01%, respectively). Among the diets, arachidonic acid (AA) showed a significantly lower value (*p* < 0.05) in fish fed the DN_0.5%_ diet (4.53 ± 0.19%). EPA was highest in the DPN_1%_, DN_1%_, and DN_0.5%_ groups (0.90 ± 0.01%, 0.89 ± 0.01%, and 0.89 ± 0.01%, respectively). DHA and the sum of EPA + DHA followed the same trend, with DC_0%_ exhibiting the lowest values (3.02 ± 0.01% and 3.86 ± 0.01%, respectively) and DP_1%_ the highest (3.67 ± 0.01% and 4.53 ± 0.01%, respectively). Total PUFA content (∑PUFAs) was highest (*p* < 0.05) in fish fed the combined diet (DPN_1%_) and the DN_1%_ diet, and lowest in DC_0%_ (Table 3).

**Table 2:** Growth performance indices (mean ± standard deviation) of Nile tilapia (*Oreochromis niloticus*) following 60 days of supplementation with: a control diet without microalgae inclusion (DC_0%_); a diet containing 10 g kg⁻¹ of *Nannochloropsis oculata* (DN_1%_); a diet containing 5 g kg⁻¹ of *N. oculata* (DN_0.5%_); a diet containing 10 g kg⁻¹ of *Phaeodactylum tricornutum* (DP_1%_); a diet containing 5 g kg⁻¹ of *P. tricornutum* (DP_0.5%_); and a diet containing 5 g kg⁻¹ of *P. tricornutum* + 5 g kg⁻¹ of *N. oculata* (DPN_1%_)

Parameters	DC_0%_	DN_1%_	DN_0.5%_	DP_1%_	DP_0.5%_	DPN_1%_	*p*-value
WG (g)	55.96 ± 13.90	55.23 ± 6.26	52.69 ± 3.88	51.19 ± 7.03	57.77 ± 9.66	60.74 ± 6.98	0.516
DWG (g)	0.93 ± 0.23	0.92 ± 0.10	0.88 ± 0.06	0.82 ± 0.12	0.96 ± 0.16	1.01 ± 0.12	0.626
SGR (% day^–1^)	5.09 ± 0.16	5.13 ± 0.52	5.14 ± 0.23	5.074 ± 0.27	5.15 ± 0.26	5.23 ± 0.23	0.984
FC	0.75 ± 0.15	0.74 ± 0.08	0.72 ± 0.05	0.78 ± 0.11	0.72 ± 0.12	0.77 ± 0.09	0.875
SR (%)	88.13 ± 22.11	95.63 ± 5.91	97.50 ± 2.04	94.37 ± 2.39	94.37 ± 2.39	97.50 ± 2.89	0.755

Note: WG: weight gain; DWG: daily weight gain; SGR: specific growth rate; FC: feed conversion; and SR: survival rate

**Table 3:** Total body fatty acid composition (%) (mean ± standard deviation) of Nile tilapia (*Oreochromis niloticus*) following 60 days of supplementation with: a control diet without microalgae inclusion (DC_0%_); a diet containing 10 g kg⁻¹ of *Nannochloropsis oculata* (DN_1%_); a diet containing 5 g kg⁻¹ of *N. oculata* (DN_0.5%_); a diet containing 10 g kg⁻¹ of *Phaeodactylum tricornutum* (DP_1%_); a diet containing 5 g kg⁻¹ of *P. tricornutum* (DP_0.5%_); and a diet containing 5 g kg⁻¹ of *P. tricornutum* + 5 g kg⁻¹ of *N. oculata* (DPN_1%_). Different letters (a, b, c) within the same row indicate significant differences according to Tukey’s post hoc test

%			Treatments				
	DC_0%_	DN_1%_	DN_0.5%_	DP_1%_	DP_0.5%_	DPN_1%_	*p*-value
C12:0	0.45 ± 0.02	0.46 ± 0.01	0.45 ± 0.01	0.45 ± 0.03	0.47 ± 0.02	0.47 ± 0.01	0.218
C13:0	0.24 ± 0.02^b^	0.28 ± 0.01^a^	0.23 ± 0.01^b^	0.24 ± 0.01^b^	0.26 ± 0.01^ab^	0.25 ± 0.01^b^	0.002
C14:0	4.22 ± 0.01^b^	4.25 ± 0.02^b^	4.26 ± 0.01_b_	4.31 ± 0.01^a^	4.19 ± 0.03^b^	4.16 ± 0.01^b^	< 0.001
C16:0	23.50 ± 0.37	23.52 ± 0.21	23.46 ± 0.16	23.35 ± 0.03	23.59 ± 0.58	23.70 ± 0.01	0.677
C17:0	1.52 ± 0.07	1.57 ± 0.01	1.62 ± 0.07	1.58 ± 0.01	1.60 ± 0.02	1.61 ± 0.01	0.053
C18:0	13.24 ± 0.49^a^	12.97 ± 0.06^ab^	12.94 ± 0.01^b^	12.93 ± 0.05^b^	12.49 ± 0.03^b^	12.55 ± 0.07^b^	< 0.001
C20:0	0.87 ± 0.01^b^	0.86 ± 0.01^b^	0.87 ± 0.01^b^	0.84 ± 0.01^b^	0.89 ± 0.01^a^	0.89 ± 0.03^a^	< 0.001
C21:0	0.52 ± 0.02^ab^	0.53 ± 0.01^ab^	0.50 ± 0.01^b^	0.53 ± 0.01a^b^	0.54 ± 0.02^a^	0.53 ± 0.01a^b^	0.012
C22:0	0.34 ± 0.01^a^	0.31 ± 0.01^b^	0.33 ± 0.01^ab^	0.31 ± 0.01^b^	0.34 ± 0.01^a^	0.35 ± 0.02^a^	< 0.001
∑SFA	44.89 ± 0.17^a^	44.75 ± 0.21^ab^	44.70 ± 0.18^b^	44.54 ± 0.08^c^	44.35 ± 0.21^c^	44.49 ± 0.06^c^	< 0.001
C14:1	1.77 ± 0.02^a^	1.77 ± 0.01^a^	1.76 ± 0.01^a^	1.73 ± 0.01^b^	1.74 ± 0.01^b^	1.72 ± 0.01^b^	< 0.001
C16:1	5.31 ± 0.05^ab^	5.24 ± 0.01^b^	5.41 ± 0.05^a^	5.37 ± 0.04^ab^	5.44 ± 0.06^ab^	5.33 ± 0.03^ab^	< 0.001
C17:1	1.00 ± 0.01^a^	0.99 ± 0.01^a^	0.93 ± 0.01^b^	0.98 ± 0.01^a^	0.99 ± 0.02^a^	0.94 ± 0.02^b^	< 0.001
C18:1	24.02 ± 0.06^a^	23.81 ± 0.02^b^	23.70 ± 0.03^b^	23.74 ± 0.21^b^	23.55 ± 0.03^b^	23.49 ± 0.01^b^	< 0.001
C20:1	0.13 ± 0.01	0.13 ± 0.01	0.13 ± 0.01	0.13 ± 0.01	0.12 ± 0.01	0.12 ± 0.01	0.224
C22:1	0.35 ± 0.01	0.42 ± 0.012	0.35 ± 0.01	0.34 ± 0.01	0.35 ± 0.01	0.35 ± 0.01	0.345
∑MUFA	32.56 ± 0.10^a^	32.37 ± 0.12^ab^	32.28 ± 0.08^b^	32.29 ± 0.18^b^	32.19 ± 0.01^b^	31.95 ± 0.04^c^	< 0.001
C18:2n-6	7.97 ± 0.04^a^	7.80 ± 0.17^b^	7.93 ± 0.09^a^	7.45 ± 0.01^c^	7.66 ± 0.27^bc^	7.98 ± 0.02^a^	< 0.001
γ-C18:3n-6	4.01 ± 0.02	4.02 ± 0.02	4.01 ± 0.01	4.00 ± 0.05	3.99 ± 0.02	4.01 ± 0.01	0.5567
α-C18:3n-3	1.56 ± 0.02^b^	1.59 ± 0.01^a^	1.56 ± 0.01^b^	1.55 ± 0.02^b^	1.57 ± 0.01^ab^	1.61 ± 0.05^a^	< 0.001
C20:3n-6	0.87 ± 0.01	0.87 ± 0.01	0.88 ± 0.01	0.87 ± 0.01	0.88 ± 0.01	0.87 ± 0.01	0.6258
C20:4n-6 (AA)	4.89 ± 0.04^a^	4.83 ± 0.01^ab^	4.53 ± 0.19^c^	4.83 ± 0.04^ab^	4.71 ± 0.11^ab^	4.87 ± 0.01^a^	< 0.001
C20:5n-3 (EPA)	0.84 ± 0.01^b^	0.89 ± 0.01^a^	0.89 ± 0.01^a^	0.86 ± 0.01^b^	0.85 ± 0.03^b^	0.90 ± 0.01^a^	< 0.001
C22:6n-3 (DHA)	3.02 ± 0.01^c^	3.40 ± 0.01^b^	3.13 ± 0.13^b^	3.67 ± 0.01^a^	3.47 ± 0.05^ab^	3.10 ± 0.08^b^	< 0.001
EPA + DHA	3.86 ± 0.01^c^	4.28 ± 0.01^b^	4.02 ± 0.14^b^	4.53 ± 0.01^a^	4.32 ± 0.08^ab^	4.00 ± 0.08^b^	< 0.001
∑PUFA	22.91 ± 0.01^c^	23.38 ± 0.01^a^	23.16 ± 0.01^b^	23.18 ± 0.01^b^	23.12 ± 0.01^b^	23.33 ± 0.01^a^	0.0014

Note: Different letters ^(a, b, c)^ within the same row indicate significant differences according to Tukey’s post hoc test

**Table 4:** Haematological parameters (mean ± standard deviation) of Nile tilapia (*Oreochromis niloticus*) after 60 days of feeding with the control diet without microalgae inclusion (DC_0%_); diet with 10 g kg⁻¹ of *Nannochloropsis oculata* (DN_1%_); diet with 5 g kg⁻¹ of *N. oculata* (DN_0.5%_); diet with 10 g kg⁻¹ of *Phaeodactylum tricornutum* (DP_1%_); diet with 5 g kg⁻¹ of *P. tricornutum* (DP_0.5%_); and diet with 5 g kg⁻¹ of *P. tricornutum* + 5 g kg⁻¹ of *N. oculata* (DPN_1%_), before and after experimental challenge with *Edwardsiella tarda*

Treatments		Parameters					
		Hb	Htc	MCV	MCH	MCHC	RBC
		(g dL^-1^)	(%)	(fL)	(g dL^-1^)	(g dL^-1^)	(×10^6^ μL^-1^)
DC_0%_	Before	7.76 ± 0.69^A^	26.80 ± 4.72^Aab^	191.21 ± 43.07^Aa^	54.31 ± 7.42^A^	29.76 ± 0.47^b^	1.39 ± 0.28^A^
	After	6.69 ± 0.84^B^	22.37 ± 2.47^Bab^	159.89 ± 15.16^Ba^	47.12 ± 6.27^B^	29.55 ± 4.29^b^	1.40 ± 0.14^B^
DN_1%_	Before	8.05 ± 0.91^A^	28.16 ± 3.81^Aa^	192.44 ± 37.27^Aa^	53.26 ± 9.18^A^	28.77 ± 3.40^b^	1.52 ± 0.27^A^
	After	6.56 ± 0.61^B^	22.266 ± 2.35^Ba^	155.76 ± 23.13^Ba^	46.95 ± 7.57^B^	29.55 ± 2.02^b^	1.46 ± 0.27^B^
DN_0.5%_	Before	7.68 ± 0.78^A^	26.47 ± 3.24^Aab^	191.3 ± 28.80^Aab^	55.42 ± 5.36^A^	29.27 ± 3.25^b^	1.37 ± 0.17^A^
	After	6.38 ± 0.56^B^	21.50 ± 1.85^Bab^	138.75 ± 14.74^Bab^	40.88 ± 4.89^B^	29.58 ± 2.14^b^	1.52 ± 0.12^B^
DP_1%_	Before	8.49 ± 1.88^A^	27.37 ± 3.86^Aab^	196.75 ± 29.67^Aa^	61.85 ± 19.13^A^	31.77 ± 10.12^a^	1.42 ± 0.20^A^
	After	6.85 ± 2.51^B^	21.87 ± 5.03^Bab^	140.37 ± 29.59^Ba^	40.32 ± 7.56^B^	29.15 ± 7.74^a^	1.44 ± 0.39^B^
DP_0.5%_	Before	7.85 ± 1.49^A^	25.82 ± 5.12^Ab^	168.70 ± 46.87^Ab^	55.42 ± 13.96^A^	36.27 ± 17.18^a^	1.50 ± 30.77^A^
	After	6.33 ± 1.72^B^	18.53 ± 2.61^Bb^	117.99 ± 26.04^Bb^	40.88 ± 16.34^B^	34.51 ± 9.58^a^	1.61 ± 0.28^B^
DPN_1%_	Before	7.9 ± 0.96^A^	27.07 ± 3.57^Aab^	200.73 ± 40.59^Aa^	57.26 ± 8.10^A^	28.83 ± 2.97^b^	1.38 ± 0.22^A^
	After	6.77 ± 1.05^B^	22.53 ± 2.28^Bab^	146.66 ± 20.32^Ba^	44.23 ± 8.41^B^	30.16 ± 4.61^b^	1.55 ± 0.15^B^
*p-*value	Diet	0.361	0.017	0.013	0.119	0.001	0.0567
	Infect	< 0.001	< 0.001	< 0.001	< 0.001	0.819	0.007
	Inter	0.952	0.612	0.165	0.277	0.824	0.186

Different uppercase letters in the same column ^(A, B)^ indicate significant differences after the experimental infection. Different lowercase letters in the same column ^(a, b, c)^ indicate significant differences among the experimental diets. Letters ^(x, y)^ in the same column indicate significant interactions between predictors. *Hb* haemoglobin; *Htc* haematocrit; *MCV* mean corpuscular volume; *MCH* mean corpuscular haemoglobin; *MCHC* mean corpuscular haemoglobin concentration; *RBC* erythrocytes; Infect: infection; *Inter* interaction

### Haematoimmunological analyses

Haemoglobin, erythrocyte count, and mean corpuscular haemoglobin (MCH) were significantly higher (*p* < 0.05) in all treatments prior to the experimental infection. A similar pattern was observed for haematocrit percentage, with additional differences among diets (*p* < 0.05), highlighting the DN_1%_ group as having the highest value. Likewise, mean corpuscular volume (MCV) varied among treatments and in response to infection (*p* < 0.05), with the lowest values recorded in the DP_0.5%_ group. Mean corpuscular haemoglobin concentration (MCHC) was higher (*p* < 0.05) in groups supplemented exclusively with *P. tricornutum*, irrespective of concentration, compared to the other groups. Glucose levels were higher during the pre-infection period (*p* < 0.05), with the lowest concentrations (*p* < 0.05) observed in the groups fed exclusively with *P. tricornutum*, irrespective of concentration. A significant interaction (*p* < 0.05) between treatment and experimental challenge was noted, particularly in the DP_1%_ and DP_0.5%_ groups post-infection, compared to DN_1%_ pre-infection.

All groups exhibited a significant increase (*p* < 0.05) in leukocyte counts following the experimental infection. The DP*1%* diet notably modulated the leukocyte response, promoting significant increases (*p* < 0.05) in total WBC, thrombocytes, lymphocytes, monocytes, and neutrophils, particularly in comparison with the control group (DC_0%_). A significant interaction between factors (*p* < 0.05) was observed for all these leukocytes, except neutrophils, indicating a distinct pattern between DC_0%_ pre-infection and DP_1%_ post-infection. Basophil counts were also significantly higher (*p* < 0.05) following infection (Tables 4 and 5).

**Fig. 1:**
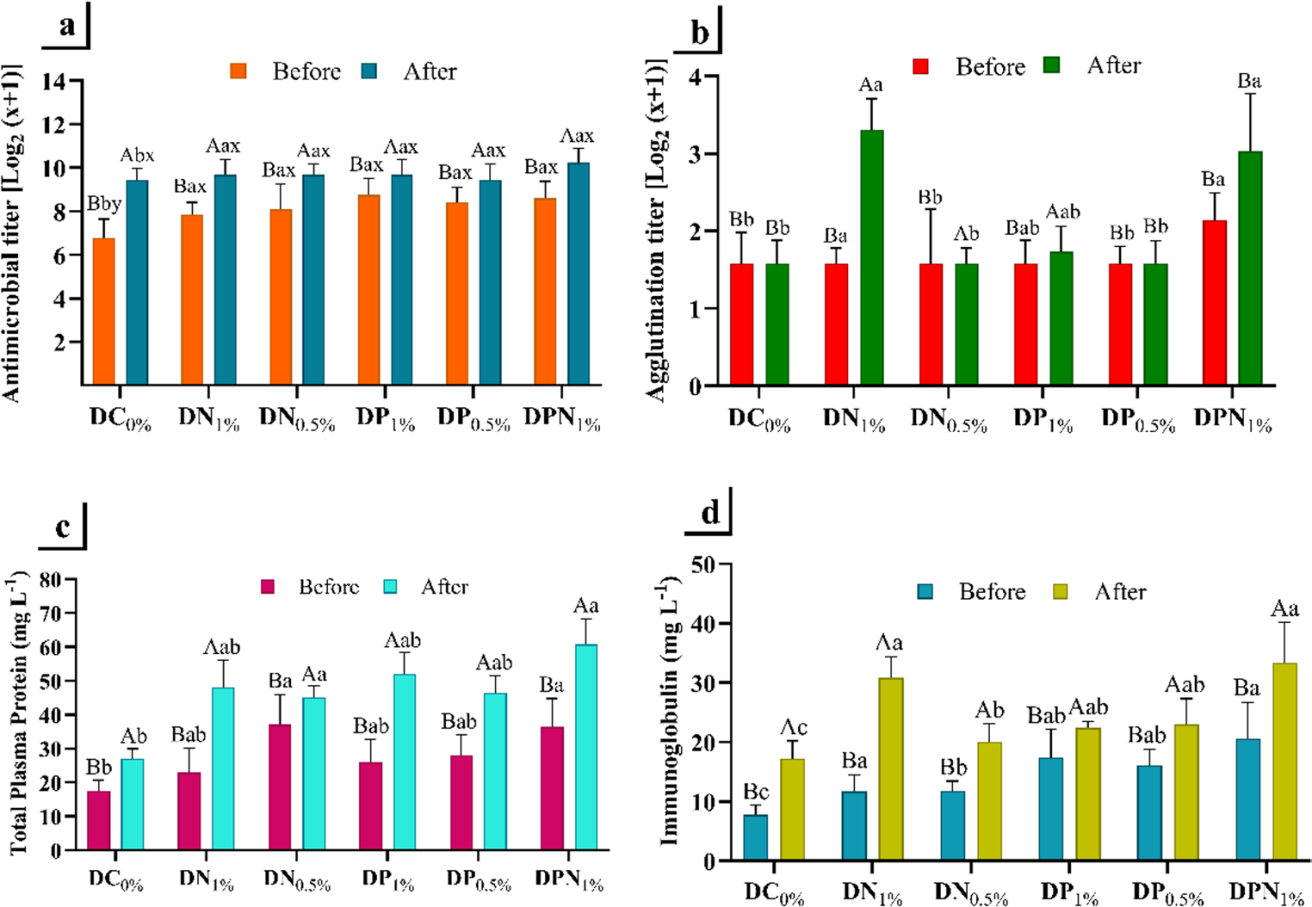
Immunological parameters of Nile tilapia (*Oreochromis niloticus*) following 60 days of dietary supplementation with the marine microalgae *Nannochloropsis oculata* and *Phaeodactylum tricornutum*. Antimicrobial (**a**) and agglutination (**b**) titres, total plasma protein (**c**), and immunoglobulin (**d**) levels in the plasma of Nile tilapia after supplementation with: a control diet without microalgae inclusion (DC_0%_); a diet containing 10 g kg⁻¹ of *N. oculata* (DN_1%_); a diet containing 5 g kg⁻¹ of *N. oculata* (DN_0.5%_); a diet containing 10 g kg⁻¹ of *P. tricornutum* (DP_1%_); a diet containing 5 g kg⁻¹ of *P. tricornutum* (DP_0.5%_); and a diet containing 5 g kg⁻¹ of *P. tricornutum* + 5 g kg⁻¹ of *N. oculata* (DPN_1%_), followed by experimental challenge with *Edwardsiella tarda*. Different uppercase letters ^(A, B)^ indicate significant differences after infection and different lowercase letters ^(a, b)^ indicate significant differences among treatments. Different letters ^(x, y)^ indicate a significant interaction between predictors

Antimicrobial titers were higher following the experimental infection, with significant differences observed between the control diet (DC_0%_) and the other diets (*p* < 0.05). Additionally, a significant interaction (*p* < 0.05) between predictors (diet × experimental infection) was noted (Fig. 1a). For agglutination titers, no interaction between predictors was observed (*p* > 0.05), although significant differences were detected after infection and among diets (*p* < 0.05), particularly between DN_0.5%_ and DPN_1%_ (Fig. 1b). Total plasma protein (TPP) and immunoglobulin (Ig) concentrations were significantly higher (*p* < 0.05) following the experimental infection. Regarding the experimental diets, TPP levels were higher (*p* < 0.05) in the DPN_1%_ and DN_0.5%_ groups, and lower (*p* < 0.05) in the control group (Fig. 1c). Ig levels were highest (*p* < 0.05) in the DPN_1%_ group and lowest (*p* < 0.05) in the DC_0%_ and DN_0.5%_ groups (Fig. 1d).

**Table 5:** Haematological parameters (mean ± standard deviation) of Nile tilapia (*Oreochromis niloticus*) after 60 days of feeding with the control diet without microalgae inclusion (DC_0%_); diet with 10 g kg⁻¹ of *Nannochloropsis oculata* (DN_1%_); diet with 5 g kg⁻¹ of *N. oculata* (DN_0.5%_); diet with 10 g kg⁻¹ of *Phaeodactylum tricornutum* (DP_1%_); diet with 5 g kg⁻¹ of *P. tricornutum* (DP_0.5%_); and diet with 5 g kg⁻¹ of *P. tricornutum* + 5 g kg⁻¹ of *N. oculata* (DPN_1%_), before and after experimental challenge with *Edwardsiella tarda*

Treatments		Parameters						
		Glucose	WBC	Thr	Lym	Mon	Neu	Bas
		(mg dL^− 1^)	(× 10^3^ µL^− 1^)	(×10^3^ µL^− 1^)	(×10^3^ µL^− 1^)	(×10^3^ µL^− 1^)	(× 10^3^ µL^− 1^)	(× 10^3^ µL^− 1^)
DC_0%_	Before	31.2 ± 5.50^Abxy^	99.64 ± 28.31^Bby^	4.20 ± 2.80^Bcy^	88.93 ± 28.80^Aby^	8.33 ± 2.83^Acy^	1.97 ± 0.92^Ac^	2.30 ± 1.12^A^
	After	20.85 ± 9.51^Bbxy^	133.91 ± 32.06^Abxy^	12.97 ± 5.28^Acxy^	108.96 ± 27.72^Bbxy^	18.81 ± 5.28^Bcxy^	4.61 ± 2.72^Bc^	5.11 ± 2.39^B^
DN_1%_	Before	37.25 ± 11.13^Aaxy^	127.57 ± 36.94^Babxy^	10.16 ± 4.80^Bbxy^	91.48 ± 30.07^Aaxy^	16.78 ± 8.00^Abxy^	4.63 ± 2.69^Ab^	3.13 ± 1.14^B^
	After	21.00 ± 6.13^Baxy^	143.32 ± 36.98^Aabxy^	14.81 ± 6.36^Abxy^	121.16 ± 30.71^Baxy^	27.35 ± 6.57^Bbxy^	4.80 ± 2.26^Bb^	7.09 ± 4.24^A^
DN_0.5%_	Before	33.8 ± 8.26^Aaxy^	119.84 ± 27.22^Baxy^	11.18 ± 5.65^Babxy^	100.26 ± 26.27^Aaxy^	15.41 ± 4.98^Abxy^	3.36 ± 1.77^Ab^	7.52 ± 2.47^B^
	After	27.67 ± 8.02^Baxy^	170.77 ± 32.61^Aaxy^	24.96 ± 9.58^Aabxy^	127.20 ± 30.50^Baxy^	36.64 ± 8.59^Bbxy^	6.18 ± 3.38^Bb^	9.08 ± 3.46^A^
DP_1%_	Before	29.05 ± 6.53^Aby^	124.83 ± 30.11^Baxy^	9.47 ± 3.90^Bbxy^	112.080 ± 27.54^Aaxy^	10.55 ± 4.70^Abxy^	1.92 ± 0.81^Ac^	2.84 ± 1.17^B^
	After	17.73 ± 4.22^Bby^	159.55 ± 30.13^Aaxy^	16.75 ± 6.80^Aaxy^	127.20 ± 38.86^Baxy^	32.29 ± 13.45^Bbxy^	4.45 ± 1.71^Bc^	5.29 ± 1.42^A^
DP_0.5%_	Before	30.68 ± 7.63^Aby^	139.10 ± 41.13^Baxy^	11.84 ± 5.02^Babxy^	114.68 ± 43.81^Aaxy^	15.91 ± 6.84^Abxy^	2.60 ± 1.42^Ab^	3.14 ± 1.00^B^
	After	18.93 ± 5.28^Bby^	174.25 ± 35.66^Aaxy^	19.92 ± 4.28^Aabxy^	123.71 ± 31.77^Baxy^	43.24 ± 11.2^Bbxy^	6.51 ± 2.11^Bb^	8.01 ± 3.20^A^
DPN_1%_	Before	32.40 ± 10.30^Aax^	129.06 ± 27.64^Baxy^	14.34 ± 6.04^Baxy^	105.81 ± 18.07^Aaxy^	23.26 ± 7.10^Aaxy^	5.09 ± 1.36^Aa^	5.30 ± 1.26^B^
	After	26.33 ± 5.69^Bax^	220.54 ± 62.79^Aax^	27.42 ± 6.58^Acx^	135.39 ± 41.44^Bax^	72.42 ± 19.18^Bax^	6.67 ± 2.86^Ba^	9.42 ± 2.50^A^
*p*-value	Diet	< 0.001	< 0.001	< 0.001	0.023	< 0.001	< 0.001	0.082
	Infect	< 0.001	< 0.001	< 0.001	0.009	< 0.001	< 0.001	0.012
	Inter	< 0.001	< 0.001	< 0.001	0.001	< 0.001	0.063	0.528

Note: Different uppercase letters in the same column ^(A, B)^ indicate significant differences after the experimental infection. Different lowercase letters in the same column ^(a, b, c)^ indicate significant differences among the experimental diets. Letters ^(x, y)^ in the same column indicate significant interactions between predictors. WBC: total leukocytes; Thr: thrombocytes; Lym: lymphocytes; Mon: monocytes; Neu: neutrophils; Bas: basophils; Infect: infection; Inter: interaction

### Experimental challenge

**Fig. 2:**
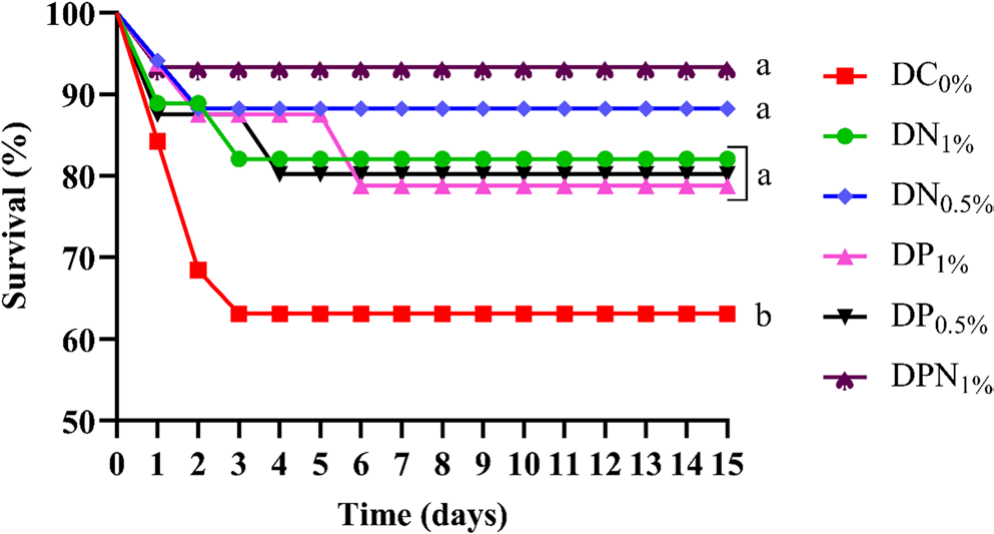
Kaplan–Meier curves depicting the probability of survival of Nile tilapia (*Oreochromis niloticus*) following experimental challenge with *Edwardsiella tarda*. Prior to the challenge, the fish were fed for 60 days with: a control diet without microalgae inclusion (DC_0%_); a diet containing 10 g kg⁻¹ of *Nannochloropsis oculata* (DN_1%_); a diet containing 5 g kg⁻¹ of *N. oculata* (DN_0.5%_); a diet containing 10 g kg⁻¹ of *Phaeodactylum tricornutum* (DP_1%_); a diet containing 5 g kg⁻¹ of *P. tricornutum* (DP_0.5%_); and a diet containing 5 g kg⁻¹ of *P. tricornutum* + 5 g kg⁻¹ of *N. oculata* (DPN_1%_). Different lowercase letters (**a, b**) indicate significant differences among treatments

Following the experimental challenge, fish fed the control diet (DC_0%_) exhibited the lowest survival rate (65.00 ± 2.00%), which was significantly different (*p* < 0.05) from the other diets. Survival rates in the remaining groups exceeded 70%, with the combined diet (DPN_1%_) showing the highest survival at 91.67 ± 1.58% (Fig. 2).

## Discussion

The present study investigated the effects of dietary supplementation with the marine microalgae *N. oculata* and *P. tricornutum* in Nile tilapia. The freeze-dried biomass of these microalgae provided several physiological benefits when included in the diets, both in terms of the fillets’ nutritional value and the overall health of the fish. The findings indicate that these microalgae provide essential nutrients, antioxidants and bioactive compounds that could enhance fish health, similar to other plant- and bioactive-based feed additives (Kusi et al. 2025; Islam et al. 2025), thereby promoting sustainable aquaculture practices.

The results regarding phenolic compounds, flavonoids, and antioxidant potential in the experimental diets were as expected, since the included microalgae are naturally rich in these bioactive compounds, particularly *P. tricornutum* (Zhang et al. 2021), which explains the higher concentrations observed in the DP_1%_ diet. With respect to tannins, the highest content in the DPN_1%_ diet can be attributed to the combined contributions of both *N. oculata* and *P. tricornutum*. This is supported by the observation that the DN_1%_ and DP_1%_ diets, which received the highest individual inclusion levels (1%), did not differ significantly from DPN_1%_.

These findings are noteworthy because phenolic compounds, as reported by Cichoński and Chrzanowski (2022), are associated with antimicrobial and immunomodulatory activities, as well as other health-promoting effects. Flavonoids, in turn, are recognised as key contributors to antioxidant capacity, playing a central role in the reduction and neutralisation of free radicals (Zhou et al. 2023). Therefore, the presence of these compounds in the experimental diets reinforces both their nutritional potential and their capacity to modulate oxidative processes. Importantly, these bioactive compounds remained available even after feed processing steps such as pelleting, drying, and storage, emphasising the stability and functional relevance of microalgae in aquafeeds.

Fatty acids are essential nutrients for fish, playing key roles in physiological processes, reproduction, and growth (Thiruvasagam et al. 2024). In this context, marine microalgae have been proposed as promising feed additives in aquaculture diets due to their high content of bioactive compounds. In the present study, although the highest individual concentrations of some saturated fatty acids (SFAs) were observed in the DP_0.5%_ group, the total ∑SFAs content was highest in the control group (DC_0%_) due to its elevated stearic acid concentration. Among SFAs, stearic acid was the second most abundant, after palmitic acid, whose levels remained stable across the different diets.

For MUFAs, a similar pattern was observed. Both individual concentrations (except for palmitoleic acid) and total ∑MUFAs were higher in the DC_0%_ group, primarily driven by oleic acid, the predominant MUFA. In contrast, the lowest concentrations were recorded in the groups supplemented with *P. tricornutum* (DP_1%_) or the combination of both microalgae (DPN_1%_). These findings are consistent with Matos et al. (2019), who reported palmitic and oleic acids as the main representatives of SFAs and MUFAs, respectively, in freshwater fish species. Conversely, the highest levels of long-chain polyunsaturated fatty acids (LC-PUFAs), such as DHA, EPA, and their sum (DHA + EPA), were found in diets containing 1% microalgae or the combination of both species. This likely reflects the high PUFA content in *N. oculata* (Matsui et al. 2020; Zahran et al. 2023)d *tricornutum* (Zhang et al. 2021; Kadalag et al. 2022). This pattern may also indicate a “LC-PUFA sparing effect,” whereby SFA and MUFA metabolic pathways are prioritised for energy production, as Henderson et al. (1996) highlight that SFAs and MUFAs serve as substrates for mitochondrial β-oxidation in fish. Complementarily, Turchini et al. (2011) note that including SFAs and MUFAs in marine feeds supplemented with LC-PUFAs supports productive performance while retaining DHA and EPA. These findings are economically relevant, as they suggest that lower-cost fatty acids, such as MUFAs, can meet energy requirements while preserving higher-value PUFAs for their functional and nutritional roles.

Additionally, the elevated ω-3 fatty acid content, particularly EPA and DHA, in the muscle of fish fed diets containing 1% microalgae enhances fillet nutritional quality and commercial value, given the well-recognised benefits of these compounds for human brain development and cardiovascular health (Schacky 2021). These results also underscore the potential of these microalgae as alternative sources of LC-PUFAs in tilapia nutrition. This is particularly important because, as Sarker et al. (2016) note, the use of fish oil–derived ω-3 sources in feed can render fillets more susceptible to lipid oxidation, thereby compromising quality. The findings are further supported by Zahran et al. (2023) and Sørensen et al. (2023), who observed increased total ω-3 PUFA profiles in tilapia (*O. niloticus*) fed *N. oculata* (5–10%) and in Atlantic salmon (*Salmo salar*) fed *P. tricornutum* (30%), respectively. Overall, the lipid profile in the present study follows the general trend described by Matos et al. (2019), with fatty acid concentrations in the order ∑SFAs > ∑MUFAs > ∑PUFAs.

Specific haematological analyses provide insight into physiological and pathological changes, contributing significantly to the assessment of fish health and nutritional status (Witeska et al. 2023). In the present study, elevated erythrocyte parameters prior to infection, irrespective of diet, possibly reflect a stable physiological state indicative of good health. The subsequent reduction in these parameters following the experimental challenge may be associated with an acute inflammatory response induced by infection, involving processes such as haemodilution and erythrocyte lysis. These findings validate the effectiveness of the infection model and demonstrate systemic mobilisation in response to the pathogen. Similarly, Reda et al. (2024) reported reductions in RBC, haemoglobin, and haematocrit in *O. niloticus* following *Shewanella* spp. infection, while El-Wafai et al. (2024) observed comparable decreases in RBC and haemoglobin after *Pseudomonas aeruginosa* infection.

In a recent study, Islam et al. (2025) simultaneously supplemented the diet of Nile tilapia with probiotics (1.0 mL L^–1^) and *Spirulina platensis* (50 g kg feed^–1^) and observed that the supplemented fish showed no significant differences in haemoglobin and blood glucose levels compared with non-supplemented fish. In contrast, Kusi et al. (2025) investigated the effects of diets supplemented with the phytobiotics *Curcuma longa*, *Allium sativum* and *Zingiber officinale* at 1% in the diet of Nile tilapia. They observed that haemoglobin, haematocrit, platelets, leukocytes, serum biochemistry and respiratory burst activity (RBA) were significantly improved in the treated groups compared with the control group. The variation in blood parameters of Nile tilapia observed across studies may be attributed to the distinct characteristics and mechanisms of action of the feed additives used. While the combination of probiotics with *S. platensis* tends to exert a balancing effect on the organism without causing significant haematological changes, phytobiotics contain bioactive compounds known for their anti-inflammatory, antioxidant and immunostimulatory properties, which may elicit more pronounced physiological responses (Brum et al. 2025).

In the present study, dietary effects on erythrogram parameters were evident, particularly the increased haematocrit in the DN_1%_ group and higher MCHC in the group supplemented exclusively with *P. tricornutum*. These effects may be attributed to bioactive metabolites such as phenolics, flavonoids, antioxidants, EPA, and carotenoids (e.g., fucoxanthin) (Reis et al. 2021a, b), which influence membrane fluidity (Adarme-Vega et al. 2012) and protect against oxidative stress by neutralising reactive oxygen species (ROS) generated during cellular metabolism (Chow et al. 2016). Similar findings were reported by Reis et al. 2021a, b; Manikandan et al. (2022), who observed increased haematocrit and MCHC in *Sparus aurata* fed 1% *P. tricornutum*, as well as elevated erythrogram parameters in *Labeo rohita* fed the seaweed *Halymenia dilatata* (25–50%).

Regarding glycaemia, post-infection reductions may result from increased energy demands during immune activation, as organisms regulate and store energy to prevent starvation and combat infection (Morano et al. 2020). Notably, fish supplemented exclusively with *P. tricornutum* maintained stable glucose levels, likely owing to higher fatty acid reserves utilised as energy sources during infection (Gwon et al. 2024; Thiruvasagam et al. 2024). This effect reflects improved metabolic control and reduced physiological stress, as supported by the significant diet × infection interaction observed in the DP_1%_ and DP_0.5%_ groups, indicating that dietary composition enhances metabolic resilience under immune challenge.

Leukocyte counts increased, particularly following the bacterial challenge, reflecting their central role in both innate and adaptive immune responses. Supplementation with 1% *P. tricornutum* significantly modulated leukocyte responses, elevating total WBC, lymphocytes, monocytes, neutrophils, and thrombocytes, which are crucial for coagulation and tissue repair during active infection. This suggests stimulation of haematopoiesis and enhanced immune cell mobilisation. The bioactive β-glucans in *P. tricornutum*, known to activate phagocytic cells (Carballo et al. 2019; Miao et al. 2020), likely contributed to the observed immunomodulatory effects.

The significant interaction between diet and infection, with the exception of neutrophils, indicates diet-dependent innate immune responsiveness. The control group (DC_0%_) exhibited lower performance, emphasising the functional potential of microalgae in enhancing innate immunity. Comparable results have been reported by Sánchez et al. (2023) in *Salmo salar* fed *Nannochloropsis gaditana* (5%) and *Schizochytrium* spp. (5%), and by Khani et al. (2017) in *Cyprinus carpio* Koi fed *Chlorella vulgaris* (5–70%), demonstrating increased leukocyte counts with microalgae supplementation.

Fish, like mammals, possess a complex immune system comprising both innate and adaptive components (Biller-Takahashi and Urbinati 2014). The innate immune response is rapid but non-specific, whereas the adaptive response develops more slowly, yet with greater specificity and effectiveness, mediated by antigen recognition through T and B lymphocytes. In the present study, plasma antimicrobial titres increased in all groups following experimental infection, indicating activation of the immune response against the pathogen. Diets supplemented with microalgae resulted in higher antimicrobial titres compared with the control group, mirroring the patterns observed in leukocyte counts. These findings suggest that microalgal supplementation may enhance the bactericidal capacity of plasma, as antimicrobial titres are associated with pathogen membrane disruption, protease inhibition and attenuation of bacterial toxicity (Uribe et al. 2011).

The increased plasma bactericidal activity may be attributed to bioactive compounds such as flavonoids and phenolics, which modulate humoral immune responses by promoting B-cell activation, proliferation and differentiation into antibody-secreting plasma cells. These findings are consistent with Guzmán et al. (2019), who reported the presence of antimicrobial peptides in *Tetraselmis suecica*, and Abdel-Tawwab et al. (2022), who observed enhanced serum bactericidal activity in *O. niloticus* fed *Scenedesmus quadricauda* (1.5–2.0%). A significant diet × infection interaction was detected for antimicrobial titres, particularly in the DP_1%_ and DPN_1%_ groups post-challenge compared with the control group (DC0%) pre-challenge, suggesting that microalgal supplementation potentiated the immune response through diet-modulated bioactive compounds.

Antigen agglutination, primarily mediated by lectins (Costa et al. 2024; Esteban 2024) and closely associated with immunoglobulins (Ermakov et al. 2020), was higher in groups receiving greater microalgal inclusion (DP_1%_, DN_1%_, DPN_1%_), irrespective of infection status. Total immunoglobulin (Ig) concentrations increased post-challenge in all groups, although they remained lowest in the control group (DC_0%_). Consistent with the agglutination results, the highest Ig levels were observed in DP_1%_, DN_1%_ and DPN_1%_, particularly following infection, reinforcing the role of immunoglobulins as principal agglutinins (Swain 2006). These effects likely reflect the influence of flavonoids and other antioxidants present at higher concentrations in microalgae-supplemented diets, which protect cells by scavenging free radicals (Zahra et al. 2024). Moreover, such compounds may stimulate the production of pro-inflammatory cytokines (IL-1β, IL-6, TNF-α), promote apoptosis and B-cell proliferation, and thereby enhance immunoglobulin synthesis (Jomova et al. 2025).

Post-infection increases in total plasma protein concentrations are consistent with inflammatory responses (Stosik et al. 2001; Uribe et al. 2011). The greater elevation of total plasma protein observed in the microalgae-fed groups suggests an intensified immune response, particularly in fish receiving the combined diet (DPN_1%_), indicating potential synergistic effects between *N. oculata* and *P. tricornutum*. Consistently, these immunological improvements, together with enhanced lipid and haematological profiles, were reflected in higher survival rates among microalgae-fed fish following a 15-day challenge with *Edwardsiella tarda*, with the most pronounced effect observed in the DPN_1%_ group.

Previous studies support these findings. Abdelghany et al. (2020) reported increased serum lysozyme activity, nitric oxide levels, total protein, globulin and albumin concentrations, as well as upregulated expression of IL-1β and TNF-α, in *O. niloticus* fed 5–10% *N. oculata*, together with enhanced resistance to *A. veronii*. Similarly, Ibrahim et al. (2022) observed dose-dependent increases in serum lysozyme activity, complement activity (ACH50) and IgM levels in *O. niloticus* fed microalgal blends, resulting in improved survival following *A. hydrophila* challenge. Likewise, Aulia et al. (2024) reported enhanced survival of *Oncorhynchus mykiss* fed diets containing 0.5% microalgae after infection with *Vibrio anguillarum*. These studies corroborate the immunostimulatory and prophylactic potential of dietary microalgae in aquaculture.

## Conclusion

The inclusion of *N. oculata* and *P. tricornutum* in the diet of Nile tilapia, although not significantly affecting zootechnical performance, proved promising in enhancing the incorporation of polyunsaturated fatty acids (PUFAs), particularly EPA and DHA, into muscle tissue. Moreover, dietary supplementation improved haematoimmunological parameters, resulting in increased resistance to *E. tarda* infection. Diets containing 1% *P. tricornutum* (DP_1%_) and the combined diet (DPN_1%_) demonstrated potential as effective nutritional strategies to promote health, strengthen immune function and enhance resistance to bacterial infections in farmed tilapia. These findings indicate that these microalgae have considerable potential as nutraceutical feed additives in aquaculture, contributing to sector sustainability. Nevertheless, further studies are warranted to elucidate the underlying mechanisms and to confirm long-term effects under commercial farming conditions.

## Supplementary Information

Below is the link to the electronic supplementary material.

Supplementary Material 1

Supplementary Material 2

## Data Availability

The data will be made available upon reasonable request.
