# Correction: Supplementing the diet of Nile tilapia (*Oreochromis niloticus*) with microalgae *Nannochloropsis oculata* and *Phaeodactylum tricornutum* enhanced immune function, lipid profile, and resistance to *Edwardsiella tarda* infection

**DOI:** 10.1007/s11259-026-11267-4

**Published:** 2026-05-14

**Authors:** Domickson Silva Costa, Jucimauro de Araújo Pereira Júnior, Paola Capistrano dos Santos, Maria Clara Miguel Libanori, Gracienhe Gomes dos Santos, Ana Paula de Souza, Alexandre Vaz da Silva, Cláudia Andrea Lima Cardoso, Arlene Sobrinho Ventura, Aline Brum Figueredo Ruschel, Rafael Sales, Fábio de Farias Neves, Marco Shizuo Owatari, Maurício Laterça Martins, Scheila Anelise Pereira Dutra, Cláudio Manoel Rodrigues de Melo

**Affiliations:** 1 Marine Molluscs Laboratory (LMM), Aquaculture Department, Federal University of Santa Catarina (UFSC), Florianópolis, SC Brazil; 2 Marine Shrimp Laboratory (LCM), Aquaculture Department, Federal University of Santa Catarina (UFSC), Florianópolis, SC Brazil; 3 Aquatic Organisms Health Laboratory (AQUOS), Aquaculture Department, Federal University of Santa Catarina (UFSC), Florianópolis, SC Brazil; 4 Federal Institute of Santa Catarina (IFC), Araquari, SC Brazil; 5 Center for Studies in Natural Resources, State University of Mato Grosso do Sul (UEMS), Dourados, MS Brazil; 6 Faculty of Agricultural Sciences (FCA), Federal University of Grande Dourados (UFGD), Dourados, MS Brazil; 7 Marine Fish Farming Laboratory (LAPMAR), Aquaculture Department, Federal University of Santa Catarina (UFSC), Florianópolis, SC Brazil; 8 Algabloom, Laguna, SC Brazil; 9 Algae Cultivation and Biotechnology Laboratory (LCBA), State University of Santa Catarina (UDESC), Laguna, SC Brazil

**Correction: Veterinary Research Communications**

10.1007/s11259-026-11218-z

In the original version of this article, Table 4 was published with incorrect data.

The corrected Table 4 is provided below:

Table 4: Haematological parameters (mean ± standard deviation) of Nile tilapia (*Oreochromis niloticus*) after 60 days of feeding with the control diet without microalgae inclusion (DC_0%_); diet with 10 g kg⁻¹ of *Nannochloropsis oculata* (DN_1%_); diet with 5 g kg⁻¹ of *N. oculata* (DN_0.5%_); diet with 10 g kg⁻¹ of *Phaeodactylum tricornutum* (DP_1%_); diet with 5 g kg⁻¹ of *P. tricornutum* (DP_0.5%_); and diet with 5 g kg⁻¹ of *P. tricornutum* + 5 g kg⁻¹ of *N. oculata* (DPN_1%_), before and after experimental challenge with *Edwardsiella tarda*.

**Table:** 

Treatments			Parameters											
			Hb		Htc		MCV		MCH		MCHC		RBC	
			(g dL^− 1^)		(%)		(fL)		(g dL^− 1^)		(g dL^− 1^)		(×10^6^ µL^− 1^)	
DC_0%_	Before		7.76 ± 0.69^A^		26.80 ± 4.72^Aab^		191.21 ± 43.07^Aa^		54.31 ± 7.42^A^		29.76 ± 0.47^b^		1.39 ± 0.28^A^	
	After		6.69 ± 0.84^B^		22.37 ± 2.47^Bab^		159.89 ± 15.16^Ba^		47.12 ± 6.27^B^		29.55 ± 4.29^b^		1.40 ± 0.14^B^	
DN_1%_	Before		8.05 ± 0.91^A^		28.16 ± 3.81^Aa^		192.44 ± 37.27^Aa^		53.26 ± 9.18^A^		28.77 ± 3.40^b^		1.52 ± 0.27^A^	
	After		6.56 ± 0.61^B^		22.266 ± 2.35^Ba^		155.76 ± 23.13^Ba^		46.95 ± 7.57^B^		29.55 ± 2.02^b^		1.46 ± 0.27^B^	
DN_0.5%_	Before		7.68 ± 0.78^A^		26.47 ± 3.24^Aab^		191.3 ± 28.80^Aab^		55.42 ± 5.36^A^		29.27 ± 3.25^b^		1.37 ± 0.17^A^	
	After		6.38 ± 0.56^B^		21.50 ± 1.85^Bab^		138.75 ± 14.74^Bab^		40.88 ± 4.89^B^		29.58 ± 2.14^b^		1.52 ± 0.12^B^	
DP_1%_	Before		8.49 ± 1.88^A^		27.37 ± 3.86^Aab^		196.75 ± 29.67^Aa^		61.85 ± 19.13^A^		31.77 ± 10.12^a^		1.42 ± 0.20^A^	
	After		6.85 ± 2.51^B^		21.87 ± 5.03^Bab^		140.37 ± 29.59^Ba^		40.32 ± 7.56^B^		29.15 ± 7.74^a^		1.44 ± 0.39^B^	
DP_0.5%_	Before		7.85 ± 1.49^A^		25.82 ± 5.12^Ab^		168.70 ± 46.87^Ab^		55.42 ± 13.96^A^		36.27 ± 17.18^a^		1.50 ± 30.77^A^	
	After		6.33 ± 1.72^B^		18.53 ± 2.61^Bb^		117.99 ± 26.04^Bb^		40.88 ± 16.34^B^		34.51 ± 9.58^a^		1.61 ± 0.28^B^	
DPN_1%_	Before		7.9 ± 0.96^A^		27.07 ± 3.57^Aab^		200.73 ± 40.59^Aa^		57.26 ± 8.10^A^		28.83 ± 2.97^b^		1.38 ± 0.22^A^	
	After		6.77 ± 1.05^B^		22.53 ± 2.28^Bab^		146.66 ± 20.32^Ba^		44.23 ± 8.41^B^		30.16 ± 4.61^b^		1.55 ± 0.15^B^	
*p-*value	Diet		0.361		0.017		0.013		0.119		0.001		0.0567	
	Infect		< 0.001		< 0.001		< 0.001		< 0.001		0.819		0.007	
	Inter		0.952		0.612		0.165		0.277		0.824		0.186	

Note: Different uppercase letters in the same column ^(A, B)^ indicate significant differences after the experimental infection. Different lowercase letters in the same column ^(a, b, c)^ indicate significant differences among the experimental diets. Letters ^(x, y)^ in the same column indicate significant interactions between predictors. Hb: haemoglobin; Htc: haematocrit; MCV: mean corpuscular volume; MCH: mean corpuscular haemoglobin; MCHC: mean corpuscular haemoglobin concentration; RBC: erythrocytes; Infect: infection; Inter: interaction.

The original article has been corrected.

