# Supplementary material for: Supplementing the diet of Nile tilapia (Oreochromis niloticus) with microalgae Nannochloropsis oculata and Phaeodactylum tricornutum enhanced immune function, lipid profile, and resistance to Edwardsiella tarda infection
Source: Vet Res Commun. 2026 Apr 18;50(4):275. doi: 10.1007/s11259-026-11218-z (PMC13091864; doi:10.1007/s11259-026-11218-z)
Supplement: Supplementary file 1 — Supplementary Material 1 [file 11259_2026_11218_MOESM1_ESM.pdf]

Supplementary file 1: Proximate composition of the experimental diets: control diet without microalgae inclusion ($DC_{0\%}$); diet with 10 g kg^{-1} of *Nannochloropsis oculata* ($DN_{1\%}$); diet with 5 g kg^{-1} of *N. oculata* ($DN_{0.5\%}$); diet with 10 g kg^{-1} of *Phaeodactylum tricornutum* ($DP_{1\%}$); diet with 5 g kg^{-1} of *P. tricornutum* ($DP_{0.5\%}$); and diet with 5 g kg^{-1} of *P. tricornutum* + 5 g kg^{-1} of *N. oculata* ($DPN_{1\%}$).

[1]Composition (g kg^{-1})	$DC_{0\%}$	$DN_{1\%}$	$DN_{0.5\%}$	$DP_{1\%}$	$DP_{0.5\%}$	$DPN_{1\%}$
Moisture	120	120	120	120	120	120
Digestible energy (kcal/kg)	3600	3600	3600	3600	3600	3600
Crude protein	460	460	460	460	460	460
Ether extract	80	80	80	80	80	80
Crude fiber	30	30	30	30	30	30
Ash	140	140	140	140	140	140
Phosphorus	10	10	10	10	10	10
N. oculata	0	10	5	0	0	5
P. tricornutum	0	0	0	10	5	5
**Proximate composition after microalgae inclusion (g kg^{-1})**						
Total carbohydrates	224	259	248	247	245	241
Crude fiber	33	21.8	21.5	25	24.2	25.4
Lipids	74.4	75.2	72.6	75	74.8	72.5
Ash	107.8	106.4	105.1	98.2	98	105.4
Crude protein	442.6	468	433.6	364.2	434	430

[1]Guaranteed levels of Supra Aqua Line Juvenile® feed, 1.7 mm: Vit. A (2,000 IU kg^{-1}), Vit. C (750 mg kg^{-1}), Vit. D_3 (5,000 IU kg^{-1}), Vit. E (160 IU kg^{-1}), Vit. K_3 (10 mg kg^{-1}), Vit. B_1 (20 mg kg^{-1}), Vit. B_2 (25 mg kg^{-1}), Vit. B_3 (160 mg kg^{-1}), Pantothenic acid (100 mg kg^{-1}), Vit. B_6 (20 mg kg^{-1}), Biotin (0.8 mg kg^{-1}), Folic acid (8.5 mg kg^{-1}), Vit. B12 (135 µg kg^{-1}), Niacin (160 mg kg^{-1}), Choline (1,800 mg kg^{-1}), Copper (6 mg kg^{-1}), Iron (50 mg kg^{-1}), Iodine (1.3 mg kg^{-1}), Manganese (15 mg kg^{-1}), Selenium (0.3 mg kg^{-1}), Zinc (100 mg kg^{-1}), Inositol (250 mg kg^{-1}).
