# Supplementary material for: Supplementing the diet of Nile tilapia (Oreochromis niloticus) with microalgae Nannochloropsis oculata and Phaeodactylum tricornutum enhanced immune function, lipid profile, and resistance to Edwardsiella tarda infection
Source: Vet Res Commun. 2026 Apr 18;50(4):275. doi: 10.1007/s11259-026-11218-z (PMC13091864; doi:10.1007/s11259-026-11218-z)

Supplementary file 2: Kaplan–Meier mortality curve of Nile tilapia (*Oreochromis niloticus*) challenged with *Edwardsiella tarda* (ATCC 15947) at concentrations of 2×10^8, 2×10^9, and 2×10^{10} CFU mL^{-1}, and sterile saline solution 0.65% (SSS) via intraperitoneal injection for determination of the lethal dose.

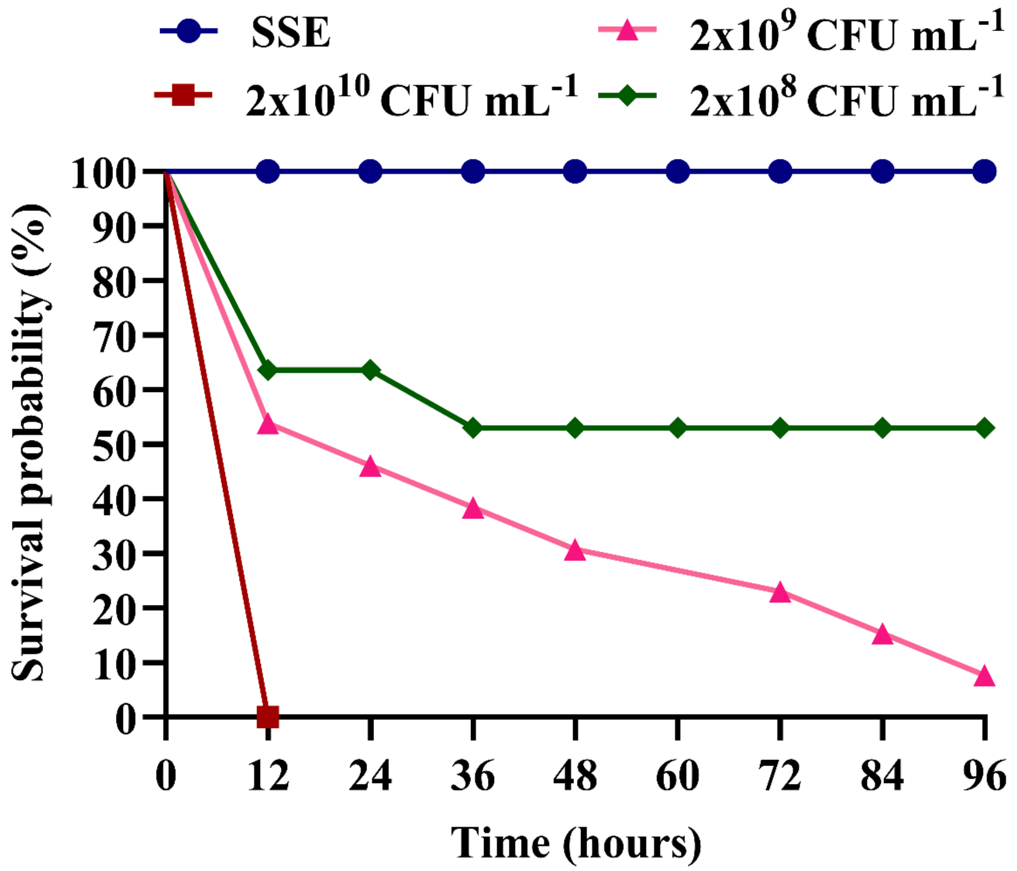

Supplement: Supplementary file 2 — Supplementary Material 2 [file 11259_2026_11218_MOESM2_ESM.pdf]
